# Supplementary material for: A regulatory pathway that selectively up-regulates elongasome function in the absence of class A PBPs
Source: eLife. 2020 Sep 8;9:e57902. doi: 10.7554/eLife.57902 (PMC7478892; doi:10.7554/eLife.57902)
Supplement: Supplementary file 1. [file elife-57902-supp1.docx]

List of primers used in this study

| **Primer number** | **Primer name** | **Sequence** |
| --- | --- | --- |
| 6395 | ponA_check_FP | CAAGACCTCTTTCCCCCTGC |
| 6396 | ponA_check_RP | CTGCACGGAATTACAAGGCG |
| 6389 | pbpD_check_FP | CGGTTTGCTGCCATGCATTA |
| 6390 | pbpD_check_RP | AGGGGAGGGGAGCATACATT |
| 6391 | pbpF_check_FP | CCGTTTCCCACAAGGTCAGA |
| 6392 | pbpF_check_RP | AGGATTGCAGCATCGACTCC |
| 6393 | pbpG_check_FP | TGCGCCACCATTTCGTGATA |
| 6394 | pbpG_check_RP | AAAACGAGCCTGCTTCCAGA |
| 7289 | ecsA_check_FP | TGATGTCCAGAACCCTGTCTC |
| 7290 | ecsA_check_RP | CCATCACCCAAAAGGACGGA |
| 7291 | ytxG_check_FP | AGCCGTTCTCATCTTCATGGG |
| 7292 | ytxG_check_RP | CACCGATGATTCCCCCGATT |
| 7692 | rasP_check_FP | AGTAGCTGTCGCTGCCTTTT |
| 7693 | rasP_check_RP | CGGAGCGTAGGAATAAGCGT |
| 7545 | sigW_check_FP | ATCGTCTAGAACTTTGACTCCGTCATGCGT |
| 8670 | sigW_check_RP | AAGATTCGGCTGCTTGGACA |
| 8671 | sigV_check_FP | TATTTTTCAGCCTGCCGCGT |
| 8672 | sigV_check_RP | TCGCTTTGACGATTTTGCCG |
| 6954 | sigI_check_FP | ATCTCTAGAAAGAGACGAAGCAAAGCGGT |
| 6957 | sigI_check_RP | ATCGGTACCCGGATGGTTTGCTGGAGTCA |
| 8664 | ftsL_check_FP | ACGGCCAACGAGGCCAGGCGTTACAGAAGTAGACGGCA |
| 8669 | ftsL_check_RP | AAGGCCTTATTGGCCTGCGCCACTTTTGTCATGCT |
| 8666 | ftsL_seq_check | AAAAGGAGGTCATCAGCCTATG |
| 7074 | rsgI_check_FP | AGCTTCCGGTCAAACAGCTT |
| 7075 | rsgI_check_RP | GTTCTGATACAGTCGCACGC |
| VP136 | rsiW_check_FP | TAAACATTCCTGTGGGGACT |
| VP137 | rsiW_check_RP | CATTTCGATTTAGCAAGTGA |
| 6588 | sigM_check_FP | CCATTGTGCCACTCCTTCAC |
| 6589 | sigM_check_RP | TGCAGTCATTTCCTGGTCGC |
| 9126 | mreBH_check_FP | AGAAATGCTCATGCCTCCAAGA |
| 9127 | mreBH_check_RP | TCATCTACTTTTCTCACAACACCT |
| 8495 | lytE_check_FP | CGATATATGAGCCGACCCCG |
| 8496 | lytE_check_RP | TAGGAGCAACCAAAACGCCT |
| 6976 | gsiB_check_FP | TCAAACAGGCGGACAGTACC |
| 6979 | gsiB_check_RP | ATGCCTTCAGAACCGTCCAG |
| 6851 | fabI_check_FP | CAACTTTGTCCGTGAAGCGG |
| 6852 | fabI_check_RP | CTCCTGCTGCTTCTCATGGT |
| 6048 | bcrC_check_FP | ACTTAACGATGCACGGGGAA |
| 6051 | bcrC_check_RP | AGTGAAGACAGCGGAAACCA |
| 8802 | walK_check_FP | AAGGCCAACGAGGCCATGCCGCGAAGTCAGAAAGA |
| 8803 | walK_check_RP | AAGGCCTTATTGGCCCTTTCACATCCCAATGCGGC |
| 8810 | walK_seq_check | AGGCGATGGAGCTTGCGAAA |
| 7088 | qRT_mreBH_FP | ATGGCAGGAAACAACGCCAC |
| 7089 | qRT_mreBH_RP | GCCGGTTGCCAATGTGGTAG |
| 7086 | qRT_lytE_FP | CATTGCCAGCCCGTCTGTTG |
| 7087 | qRT_lytE_RP | TGTACGCCGTCAGATCCTGC |
| 8726 | qRT_gyrA_FP | GGCGGCCATGCGTTATACAG |
| 8727 | qRT_gyrA_RP | GCCATACCTACCGCAATGCC |
| 7848 | ecsA-HindIII-F | ATCGAAGCTTACACTATAAGGGGAGAAACTATGTC |
| 7849 | ecsA-XbaI-R | ATCGTCTAGACCGCGACTGCCAAATATCAAG |
| 7850 | ecsAB-XbaI-R | ATCGTCTAGAGCAGCTGATTATCTGTCATTTGAT |
| 7851 | ytxG-HindIII-F | ATCGAAGCTTATCCTGCAGCAAGGAGGTTT |
| 7852 | ytxG-XbaI-R | ATCGTCTAGATGCTCATCATTATTCCTCCTCTT |
| 8499 | RasP-XmaI-RBS-F | ATCGCCCGGGTAAGGAGGCAAAATCATGTTCGTGAATACAGTTATAGCGTT |
| 8500 | RasP-SphI-R | ATCGGCATGCGGAGCGTAGGAATAAGCGT |
| 8662 | ftsL-gRNA-F | TACGTTGACAATCACTTTTTTCTC |
| 8663 | ftsL-gRNA-R | AAACGAGAAAAAAGTGATTGTCAA |
| 8664 | CRISPR-ftsL-up-F | ACGGCCAACGAGGCCAGGCGTTACAGAAGTAGACGGCA |
| 8665 | CRISPR-ftsL-up-R | CATAGGCTGATGACCTCCTTTT |
| 8666 | ftsL-mid-F | AAAAGGAGGTCATCAGCCTATG |
| 8667 | ftsL-mid-R | ATTTATTCTTTTTTGGCATTTGAATCATGCATAATCAGGAACATCATAAGGATA |
| 8668 | CRISPR-ftsL-down-F | TATCCTTATGATGTTCCTGATTATGCATGATTCAAATGCCAAAAAAGAATAAAT |
| 8669 | CRISPR-ftsL-downR | AAGGCCTTATTGGCCTGCGCCACTTTTGTCATGCT |
| 8519 | gBlock-ftsL-delta25-HA | TTAAAAGGAGGTCATCAGCCTATGAAAAGGGCTTCCATTACTCTCGGAGAAAAAGTGCTTCTTGTCCTCTTTGCTGCGGCGGTGCTCAGCGTATCGCTTTTGATCGTATCGAAGGCGTATGCGGCATATCAAACCAATATTGAGGTGCAAAAGCTTGAGGAGCAAATTTCATCCGAAAATAAGCAAATTGGTGACCTCGAAAAAAGCGTTGCTGATTTAAGCAAACCGCAGCGCATTATGGACATTGCGAAAAAGAACGGCTTGAACCTTAAAGATAAGAAAGTGAAAAACATACAGGAAGGATCAGGCCAAGGACCGGGAAGCGGACAAGGCCCTTATCCTTATGATGTTCCTGATTATGCTTGATAA |
| 9115 | sigI-HindIII-FP | CCCAAGCTTTCCCTATCAAATTAGCTATCA |
| 9116 | sigI-Xba1-RP | CTAGTCTAGATCTACTATAATCCCTCTTCTCA |
| 6461 | P_M_-up-for(HindIII) | GCGAAGCTTTGAGCCTTCGTCCTTTAGTGAC |
| 6457 | P_M_-up-rev | TTTTGCCAACCATTG**A**AC**A**TTTTCTTCTGGTGAAAAGTTTCGATT |
| 6458 | P_M_-do-for | AAATGTTCAATGGTTGGCAAAA |
| 6462 | P_M_-do-rev(BamHI) | GCGGGATCCTCATTGTTTTTGATATAGGAAG |
| 6485 | maf-for(SalI) | GCGGTCGACAGTATCTTTTTTTCTTAAGCAA |
| 6486 | maf-rev(AsiSI) | GCGGCGATCGCTCTTTCCCTTCCCGTTTATAGA |
| 7426 | Delta-Pm-rodA-up-F | AAGGCCAACGAGGCCTCTGCTGAACACAGTCACTT |
| 7427 | Delta-Pm-rodA-up-R | CGCTTTTTCAGCTACACGAAATGCGATAATGTGTTATGTTCCC |
| 7428 | Delta-Pm-rodA-down-F | GGGAACATAACACATTATCGCATTTCGTGTAGCTGAAAAAGCG |
| 7429 | Delta-Pm-rodA-down-R | AAGGCCTTATTGGCCCTCATTTGAAGCAGACACCC |
| 7430 | ProdA-deltaPm-gRNA-F | TACGCGTTTTTTAACAAATTCTAT |
| 7431 | ProdA-deltaPm-gRNA-R | AAACATAGAATTTGTTAAAAAACG |
| VP475 | SfiI PM ponA up for | AAGGCCAACGAGGCCGTCTCTCCCGGTTAACATTCTTTGCTCT |
| VP476 | SfiI pm ponA down rev | AAGGCCTTATTGGCCCGCCCTTTGTAAATCCCATTGTAGTCT |
| VP477 | Int repair frag F1 | CCAATCTG TTTTCGTTCC**A**TCTAATGAGTGG |
| VP478 | Int repair R1 | CCACTCATTAGATGGAACGAAAACAGATTGG |
| VP479 | Int repair F2 | ACGAAGGATTTTGTATAATAGGAGTCTGAATAGTGTTTTGTTCA |
| VP480 | Int repair R2 | TGAACAAAACACTATTCAGACTCCTATTATACAAAATCCTTCGT |
| VP481 | Int repair F3 | ACTGCCTTTTAAGTCTGTCATTGACCAGCC |
| VP482 | Int repair R3 | GGCTGGTCAATGACAGACTTAAAAGGCAGT |
| 9113 | MreBH_FP_HindIII | CCCAAGCTTtAAGGAGGTGAACATGTTTCAATCAACTG |
| 9114 | MreBH_RP_XbaI | CTAGTCTAGAAGAAGCAAGAAATTTGCC |
| 9140 | lytE-SmaI-FP | TCCCCCGGGAGGAGGAAAATATGAAAAAGCAAA |
| 9141 | lytE-XbaI-RP | CTAGTCTAGACAATGAACGGGTTTTCTCTAAA |
| 9130 | lacZ-check-FP | AGTCGTTTGCCGTCTGAATTTG |
| 9131 | lacZ-check-RP | TTTTGATGGACCATTTCGGCAC |
| 9132 | lacI-check-FP | TGACGGCGGGATATAACATGAG |
| 9133 | lacI-check-RP | ATGCCGGTGTCTCTTATCAGAC |
| 7082 | F-pSpachy-check | GCAACGCCAATCAGCAACGA |
| 7083 | R-pSpachy-check | AGACCTCACTAGGCACCTTA |
| 9144 | mreBH-EcorI-FP | CCGGAATTCCGGAAGGAGGTGAACATGTTTCAATCAACTG |
| 9145 | mreBH-SpeI-RP | GGACTAGTCCAAGAAGCAAGAAATTTGCC |
| 9231 | xylR-CDS-check-FP | TTCCAAATGGCGGTATTGAT |
| 9230 | mreBH-middle-check-RP | CATGGCCAATTTCCATTTTT |
| 8802 | WalK-Up-F | AAGGCCAACGAGGCCATGCCGCGAAGTCAGAAAGA |
| 8803 | WalK-Down-R | AAGGCCTTATTGGCCCTTTCACATCCCAATGCGGC |
| 8806 | WalK-Asp274Ala-up-R | TAATCGCTCCGTTTCGGTTTGTAGCGATAACGCCAGCTGTCATATAAGCAATGACAGAGG |
| 8807 | WalK-Asp274Ala-down-F | CCTCTGTCATTGCTTATATGACAGCTGGCGTTATCGCTACAAACCGAAACGGAGCGATTA |
| 8814 | sgRNA-walK-Asp274Ala-F | TACGAATCGCTCCGTTTCGGTTTG |
| 8815 | sgRNA-walK-Asp274Ala-R | AAACCAAACCGAAACGGAGCGATT |
| **Primers used for TnSeq** |  |  |
|  | Adapterbarcode501A | TTCCCTACACGACGCTCTTCCGATCTTATAGCCTNN |
|  | Adapterbarcode501B | AGGCTATAAGATCGGAAGAGCGTCGTGTAGGGAAAGAG |
|  | Adapterbarcode502A | TTCCCTACACGACGCTCTTCCGATCTATAGAGGCNN |
|  | Adapterbarcode502B | GCCTCTATAGATCGGAAGAGCGTCGTGTAGGGAAAGAG |
|  | Adapterbarcode503A | TTCCCTACACGACGCTCTTCCGATCTCCTATCCTNN |
|  | Adapterbarcode503B | AGGATAGGAGATCGGAAGAGCGTCGTGTAGGGAAAGAG |
|  | Adapterbarcode504A | TTCCCTACACGACGCTCTTCCGATCTGGCTCTGANN |
|  | Adapterbarcode504B | TCAGAGCCAGATCGGAAGAGCGTCGTGTAGGGAAAGAG |
|  | Adapterbarcode505A | TTCCCTACACGACGCTCTTCCGATCTAGGCGAAGNN |
|  | Adapterbarcode505B | CTTCGCCTAGATCGGAAGAGCGTCGTGTAGGGAAAGAG |
|  | Adapterbarcode506A | TTCCCTACACGACGCTCTTCCGATCTTAATCTTANN |
|  | Adapterbarcode506B | TAAGATTAAGATCGGAAGAGCGTCGTGTAGGGAAAGAG |
|  | Adapterbarcode507A | TTCCCTACACGACGCTCTTCCGATCTCAGGACGTNN |
|  | Adapterbarcode507B | ACGTCCTGAGATCGGAAGAGCGTCGTGTAGGGAAAGAG |
|  | Adapterbarcode508A | TTCCCTACACGACGCTCTTCCGATCTGTACTGACNN |
|  | Adapterbarcode508B | GTCAGTACAGATCGGAAGAGCGTCGTGTAGGGAAAGAG |
|  | oKrmit-Tnseq2 | CAAGCAGAAGACGGCATACGAAGCGCCTACGAGGAATTTGTATCG |
|  | oAdapterPCR | AATGATACGGCGACCACCGAGATCACACTCTTTCCCTACACGACGCTCTTCC |
